# Utilization of ex vivo tissue model to study skin regeneration following microneedle stimuli

**DOI:** 10.1038/s41598-022-22481-w

**Published:** 2022-10-27

**Authors:** Xue Liu, Rebecca Barresi, Michael Kaminer, Kun Qian, Fabienne Thillou, Michel Bataillon, I-Chien Liao, Qian Zheng, Charbel Bouez

**Affiliations:** 1 L’Oreal Research and Innovation, Clark, NJ USA; 2 Skincare Physicians, Chestnut Hill, MA USA; 3 Episkin, Lyon, France

**Keywords:** Biological techniques, Biomarkers, Health care, Medical research

## Abstract

Microneedling is a popular skin resurfacing and rejuvenation procedure. In order to develop better adjunct products for consumers, there is a scientific need to establish greater understanding of the mechanism in which microneedling stimulates regeneration within skin. The purpose of this study is to develop a physiologically relevant ex vivo tissue model which closely mimics the actual microneedling procedure to elucidate its mechanism of action. In this study, human ex vivo skin was subjected to microneedling treatment and cultured for 6 days. Histological analysis demonstrated that the ex vivo skin was able to heal from microneedling injury throughout the culture period. Microneedling treatment stimulated proliferation and barrier renewal of the skin. The procedure also increased the levels of inflammatory cytokines and angiogenic growth factors in a dynamic and time dependent fashion. The tissue demonstrated hallmark signs of epidermal regeneration through morphological and molecular changes after the treatment. This is one of the first works to date that utilizes microneedled ex vivo skin to demonstrate its regenerative behavior. Our model recapitulates the main features of the microneedling treatment and enables the evaluation of future cosmetic active ingredients used in conjunction with microneedling.

## Introduction

Minimally-invasive aesthetic procedures are popular options for consumers seeking solutions to cosmetic concerns^1^. Technological advancements in the past few decades have enabled easily accessible and affordable aesthetic options for facial skin rejuvenation and correction^2^. Amongst minimally-invasive procedures, microneedling is a popular and fast-growing cosmetic treatments^3^. Microneedling has shown effectiveness in skin rejuvenation, scar remodeling, melasma, and other pigmentary skin disorders^2,4–8^. It is achieved by using solid needle pins to puncture the epidermis (or dermis, depending on needle length) which creates microwounds that trigger a downstream wound healing cascade^9^. These microwounds can also serve as microscopic channels for large molecules to overcome the skin barrier and penetrate to deeper layers of the tissue^10^. The microneedling cosmetic procedure induces skin regeneration and provides enhanced penetration for actives in post-procedure products^11^.

Common devices on the market range from mechanical rollers to automated electric pens. Each instrument varies based on the needle length and density, in addition to needle speed (in the case of electrically powered devices). Home-use dermarollers (less than 0.5 mm needle length) have demonstrated the ability to reduce the appearance of pores and fine lines and to improve the absorption of skincare products^12^. Meanwhile, microneedling conducted in a clinical setting with an automated dermapen (needle length between 0.5 and 3.5 mm) created deeper microwounds which induced neoelastogenesis and neocollagenesis^12^. Two of the most common clinically used dermapens are the FDA-cleared Candela Exceed (Exceed, Amiea Med, MT.DERM GmbH, Berlin, Germany) and SkinPen devices (Crown Aesthetics, Dallas, TX, USA). The Candela Exceed medical microneedling device is a system that uses adjustable needle speeds and penetration depths to treat acne scarring and wrinkles^13^. Similarly, the SkinPen is indicated for the treatment of acne scars and induction of skin rejuvenation. It has been clinically demonstrated that the use of a dermapen with 1.0 mm needle length significantly improved the skin texture and tightness^14^.

Further understanding of the mechanism of action behind microneedling is necessary not only to help improve patient care, but may also provide opportunities to regulate molecular pathways specific to microneedling treatments^15^. It also opens opportunities of pairing treatments with specific cosmetic actives to augment procedure outcomes and reduce adverse effects^11^. Therefore, the purpose of this study is to develop a physiologically relevant human ex vivo tissue model that closely mimics microneedling procedure conditions to elucidate its mechanism of action.

## Materials and methods

### Ex vivo tissue culture and microneedling procedure

Fresh ex vivo skin (10 donors between 37 and 64 years old, female, 4 African American donors, 4 Caucasian donors, and 2 Hispanic donors) were acquired one day post-abdominoplasty procedure (BioIVT, Westbury, NY, USA). The study was approved by the WCG IRB (IRB tracking number: 20180798). The use of human abdominoplasty tissue post-corrective procedure has received informed consent from all donors. All experiments were performed in accordance with relevant guidelines and regulations.

Upon reception, the hypodermis layer was removed, and the tissue underwent two Phosphate Buffered Saline (PBS) washes to clean the blood residue. Tissue was then subjected to treatment using a microneedling pen (36-pin needles, Dr. Pen A6 Cartridges Tips, Dr. Pen Inc, San Jose, CA, USA) with a needle length of 1.5 mm. Tissue was subjected to 5-passes of the microneedle^16,17^. Following treatment, 1.2 cm diameter skin biopsies were created (a minimum of N = 3 per condition) and cultured at air–liquid interface. Skin explants not subjected to microneedling served as the untreated control group. All samples were cultured in Dulbecco’s Modified Eagle’s Medium (650µL per well, DMEM with 10% Fetal Bovine Serum and 1% Penicillin–Streptomycin) at 37 °C and 5% CO_2._ The media was changed every other day with the exception of weekends. Following a 6 day culture period, all biopsies were processed for histological and immunohistochemical analysis.

### Histological and immunohistochemical staining

Ex vivo skin tissues were processed for haematoxylin and eosin (H&E) staining (Reveal Biosciences, San Diego, CA, USA) using a standard protocol. Immunohistochemical staining was performed using a Leica Bond automated immunostainer (Reveal Biosciences, San Diego, CA, USA). Tissue samples were fixed in 10% formalin, processed and embedded in paraffin wax. Heat induced antigen retrieval was performed using Leica Bond Epitope Retrieval Buffer and endogenous peroxidase was blocked for 20 min. This was followed by blocking non-specific antibody binding using a Novolink Protein for 30 min. The sections were then incubated with the primary antibody (Mouse Monoclonal to anti-Filaggrin, 1:200 dilution, Cat# MA5-13440, Thermofisher, Waltham, MA, USA). The anti-Filaggrin was detected and visualized with 3′3 diaminobenzidine (DAB). All sections were then counterstained with a haematoxylin nuclear stain. The primary antibody was applied to tissue stained against Ki67 overnight at 4 °C (anti-Ki67, Rabbit Monoclonal, 1:100 dilution, Cat# ab16667, Abcam, Waltham, MA, USA). Donkey anti-rabbit IgG Alexa Fluor 647 was applied for 60 min followed by DAPI nuclear counterstain. The sections stained against Transglutaminase-1 (TGM-1) were incubated with the TGM-1 primary antibody (anti-TGM-1, Mouse Monoclonal, 1:100 dilution, Cat# pab0060, Covalab, Bron, France). The images were acquired with EVOS XL core microscope and a fluorescent microscope (Leica DM500, Wetzlar, Germany). Staining intensity was reported by calculating the optical density of staining using MATLAB R2020a. Image analysis for Ki67 and TGM-1 antibodies were calculated using 2 fields of view from 2 biological samples with a total 2 donors per antibody. For Filaggrin, 2 fields of view per sample, 3 biological samples per donor, 5 donors with N = 30 in total were used for calculations. All the image analysis for filaggrin samples used the same parameters.

### Multiplex cytokine assay and ELISA

In this study, supernatant (media secreted from the explants) from the Day 1 and Day 6 timepoint was collected to evaluate 20 cytokines and chemokines. Media from the Day 6 timepoint was accumulated from the most recent media change on Day 4 of culture. Multiplex cytokine array (Inflammation 20-Plex Human ProcartaPlex™ Paneld, Thermofisher, Waltham, MA, USA) was performed using the Luminex™ MAGPIX™ Instrument System (Thermofisher, Waltham, MA, USA) according to manufacturer instructions. TGF-β was evaluated according to manufacturer instructions with commercial ELISA kits (TGF-β, Cat# DY240, R&D Systems, Minneapolis, MN, USA). Due to the variation in size of tissue from different donors, the sample size from one donor for each treatment condition varied between 3 and 6 biopsies.

### Measurement of transepithelial electrical resistance

Transepithelial electrical resistance (TEER) is commonly used to measure the tight junction integrity of an epithelial monolayer and assess skin barrier function^18^. In order to measure across the ex vivo skin, a biocompatible adhesive was used to seal the gap between the skin and the wall of the transwell (Kwik-Cast Sealant, World Precision Instrument, Sarasota, FL, USA). Following the sealing of the tissue, TEER measurements were acquired on Day 0 right after the application of microneedling procedure, and then measured until Day 6 (EVOM2, World Precision Instrument, Sarasota, FL, USA). During measurements, the transwell was filled with 500µL DPBS(Thermofisher, Cat# 14190144, Waltham, MA, USA) in the apical region and 2 mL of DPBS in the basal region. The contribution of the transwell alone was measured and subtracted from the sample values. TEER value in Ω cm^2^ was obtained by multiplying the electrical resistance with skin surface area.

### Determination of gene expression in the skin samples

The skin samples used for quantitative polymerase chain reaction (qPCR), were fixed in RNA*later* (Sigma, Cat# R0901, St. Louis, MO, US) for 2 h at room temperature and stored at − 80 °C. Three individual skin explants from 1 donor were used for each treatment group. The samples were disrupted by bead-beating in lysis buffer and RNA was isolated using a Qiagen RNeasy kit (Qiagen, Cat# 74106, Hilden, Germany) in accordance with the manufacturer’s protocol. RNA concentration and purity were determined using a Nanodrop 2000 spectrophotometer (Thermofisher, Waltham, MA, USA). cDNA was generated from 230 ng RNA per sample using a High-Capacity cDNA Synthesis Kit (Thermofisher, Cat# 4368814, Waltham, MA, USA) according to the manufacturer’s instructions for Taqman single tube assay processing. qPCR reactions were run using validated Taqman gene expression assays. The plates were run using the Life Technologies QuantStudio 12K Flex instrument. Each gene was assayed in duplicate. Primers for K5, K14, NOTCH1, PCNA, and PPARD were used according to Applied Biosystems protocol. Results were normalized using reference gene PPIA, obtained from Applied Biosystems. Final results were interpreted as normalized RQ values to the control treatment group.

### Statistical analysis

Data was assessed using Student’s t-test, and two-way ANOVA with multiple comparison Holm-Sidak test. The differences between groups were compared using Graphpad Prism 9.0.1 (GraphPad Software Inc., La Jolla, CA, USA).

## Results

In this study, the effects of the microneedling procedure on human skin tissue ranging from molecular to tissue level were investigated. The results illustrated the changes in tissue healing morphology, barrier function markers, pro-inflammatory cytokines and growth factors involved in different phases of wound healing, and epidermal homeostasis gene expression.

### Morphological and barrier function evaluation of ex vivo skin tissue

**Figure 1:**
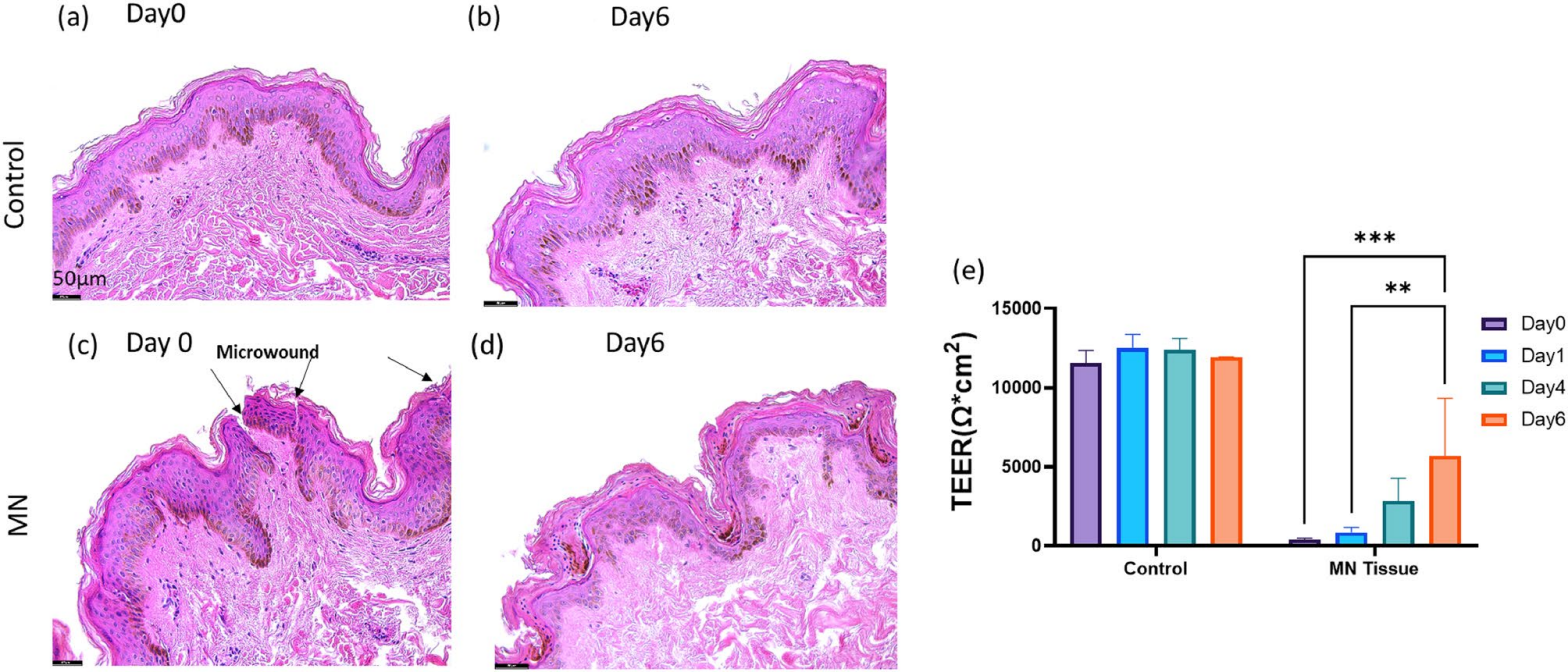
Characterization of ex vivo skin tissue. H&E staining of the control group tissue (**a**,**b**) and microneedle (MN) treated tissue on Day 0 and Day 6 (**c**,**d**). (**e**) The values of TEER which indicate tight junction barrier function integrity of ex vivo tissue on Day 0 and Day 6 (N = 4 biological samples, 1 donor). TEER was statistically tested using two-way ANOVA with multiple comparison Holm-Sidak test. Statistical differences are noted as **P ≤ 0.01, ***P ≤ 0.001. Scale bar 50 µm.

The morphology of the tissue was evaluated using H&E staining. As shown in Fig. 1c, micropunctures throughout the epidermis were observed after 5 passes of microneedling (MN) treatment. In 1.5 mm length needle conditions, the needles reached the dermal papillary region. Pinpoint bleeding, which is typically used as a reliable end point for the procedure in clinics, was observed in the tissue (Supplementary Fig. 1f)^6^. After 6 days of culture, the control samples (Fig. 1b) maintained similar morphology compared the Day 0 samples (Fig. 1a). The wound in the MN group (Fig. 1d) showed a healing process with keratinocytes covering the injured area. The effect of different length and passes on creating micropunctures within the skin tissue was also evaluated (Supplementary Fig. 1). It illustrated that the 0.25 mm length needle can penetrate through the upper layer epidermis, while both 1.0 and 1.5 mm length needles reach to the dermal layer.

Skin barrier function after microneedling was investigated using transepithelial electrical resistance (TEER) and immunochemical staining. TEER values of the tissue treated with MN dropped significantly immediately following the procedure, indicating that the skin barrier is compromised (Fig. 1e). After 6 days in culture, the TEER value of MN tissue increased from 393.7 ± 93.2 to 5685 ± 3659.5 Ω cm^2^. However, the barrier function of microneedled tissues was not fully restored when it was compared to the control group (11,936.7 ± 11.6 Ω cm^2^), indicating the tissue was still undergoing phases of wound repair.

### Characterization of the epidermal regeneration with microneedling procedure

**Figure 2:**
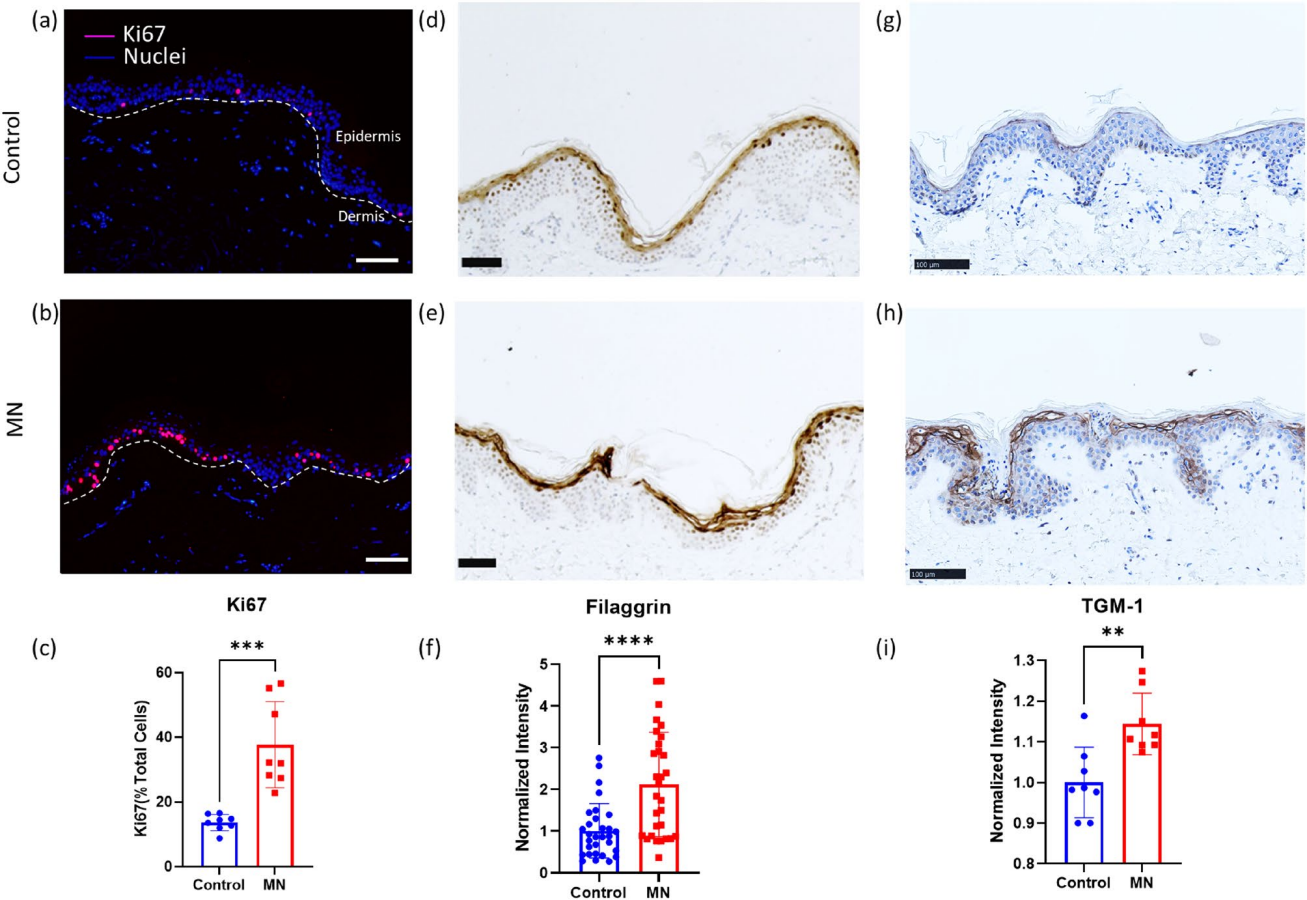
Epidermal regeneration of ex vivo tissue with and without microneedling procedure. Keratinocytes from control (**a**) and MN (**b**) groups express the proliferation marker Ki67 on Day 6 (pink, N = 8, 2 fields of view from 2 biological samples per treatment, 2 donors). The nuclei were stained with DAPI (blue). White dashed lines indicate the dermal-epidermal boundary. (**c**) Basal cell proliferation level was quantified based on the Ki67 positive cells in multiple regions of the epidermis. (**d**,**e**) Immunostaining for differentiation marker Filaggrin in control and MN treatment groups. (**f**) Normalized Filaggrin intensity of control and MN groups (N = 30, 2 fields of view per sample, 3 biological samples per donor, 5 donors). (**g**,**h**) Immunostaining for differentiation marker TGM-1 in control and MN treatment groups. (**i**) Normalized TGM-1 intensity of control and MN groups (N = 8, 2 fields of view from 2 biological samples per treatment, 2 donors). Student t-test, *P ≤ 0.05, **P ≤ 0.01, ***P ≤ 0.001, ****P ≤ 0.0001. Scale bar 100 µm.

Immunostaining with anti-Ki67 antibody (Fig. 2a–c) confirmed the proliferation of keratinocytes from tissue harvested on Day 6. The number of positively stained Ki67 cells was significantly higher in the MN treated tissue (37.7 ± 13.2%) compared to the control group (13.7 ± 2.5%). This suggests that the MN procedure stimulates keratinocyte proliferation as a repair mechanism around the wound sites^5^. Quantitative PCR data suggests an upregulation of K5 and K14 (Supplementary Fig. 2), which further supports that the procedure stimulated keratinocyte proliferation in the basal layer of the epidermis.

In addition, an upregulation of Filaggrin (Fig. 2d–f) and TGM-1 (Fig. 2g–i) was also observed in the MN treated group. The activity of Filaggrin and TGM-1 is crucial to the cornified envelope assembly process and hence explains the increased epidermal thickness after microneedling in human subjects in previous clinical and animal studies ^5,7^.

### Evaluation of wound healing phases

**Figure 3:**
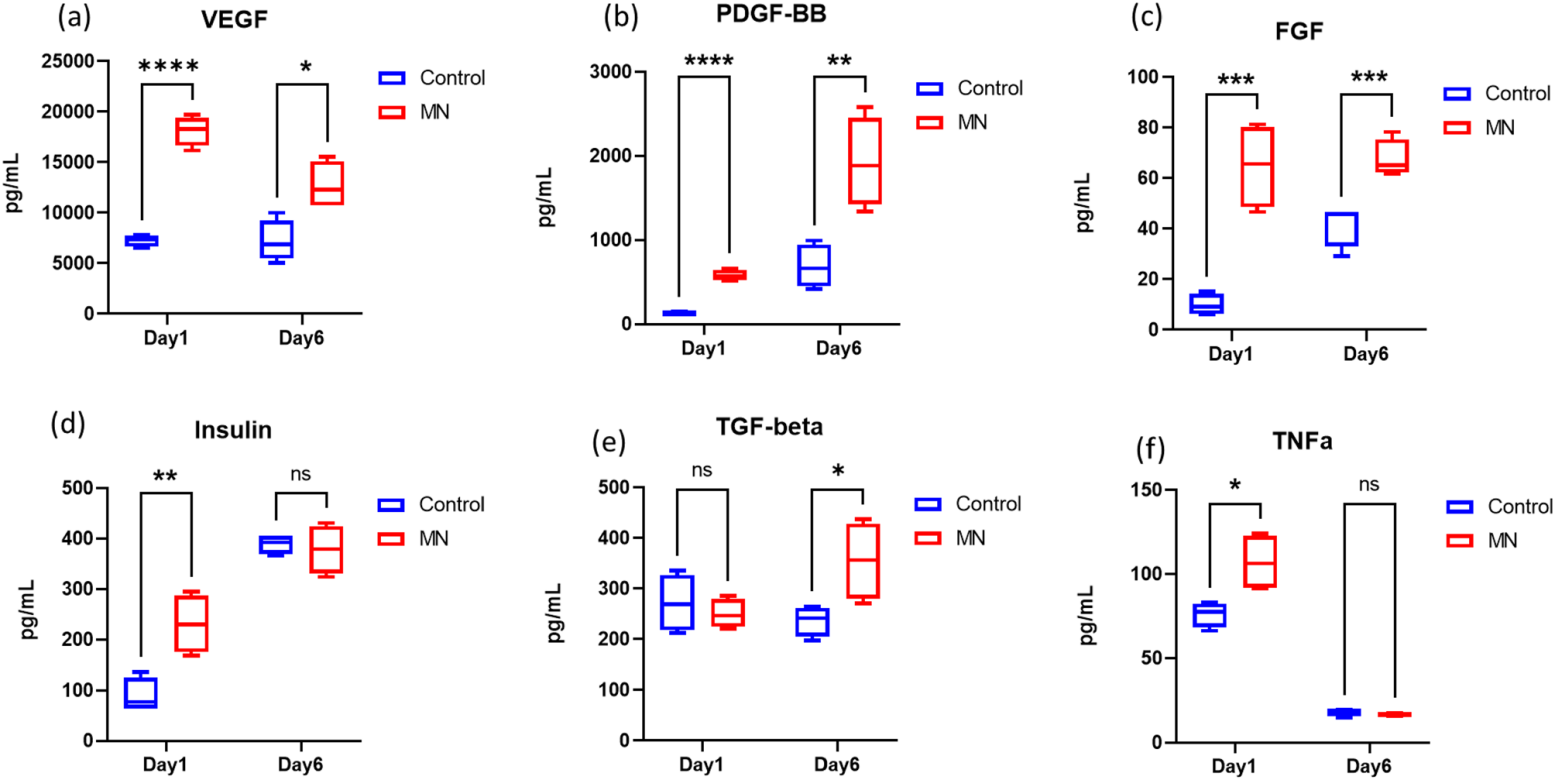
Quantification of growth factors in the ex vivo tissue. (**a**–**f**) The expression of growth factors was measured in pg/mL using a multiplex cytokine array. The medium was collected on Day 1 and Day 6 (N = 4, 2 biological samples per donor, 2 donors). Student t-test, *P ≤ 0.05, **P ≤ 0.01, ***P ≤ 0.001, ****P ≤ 0.0001.

**Figure 4:**
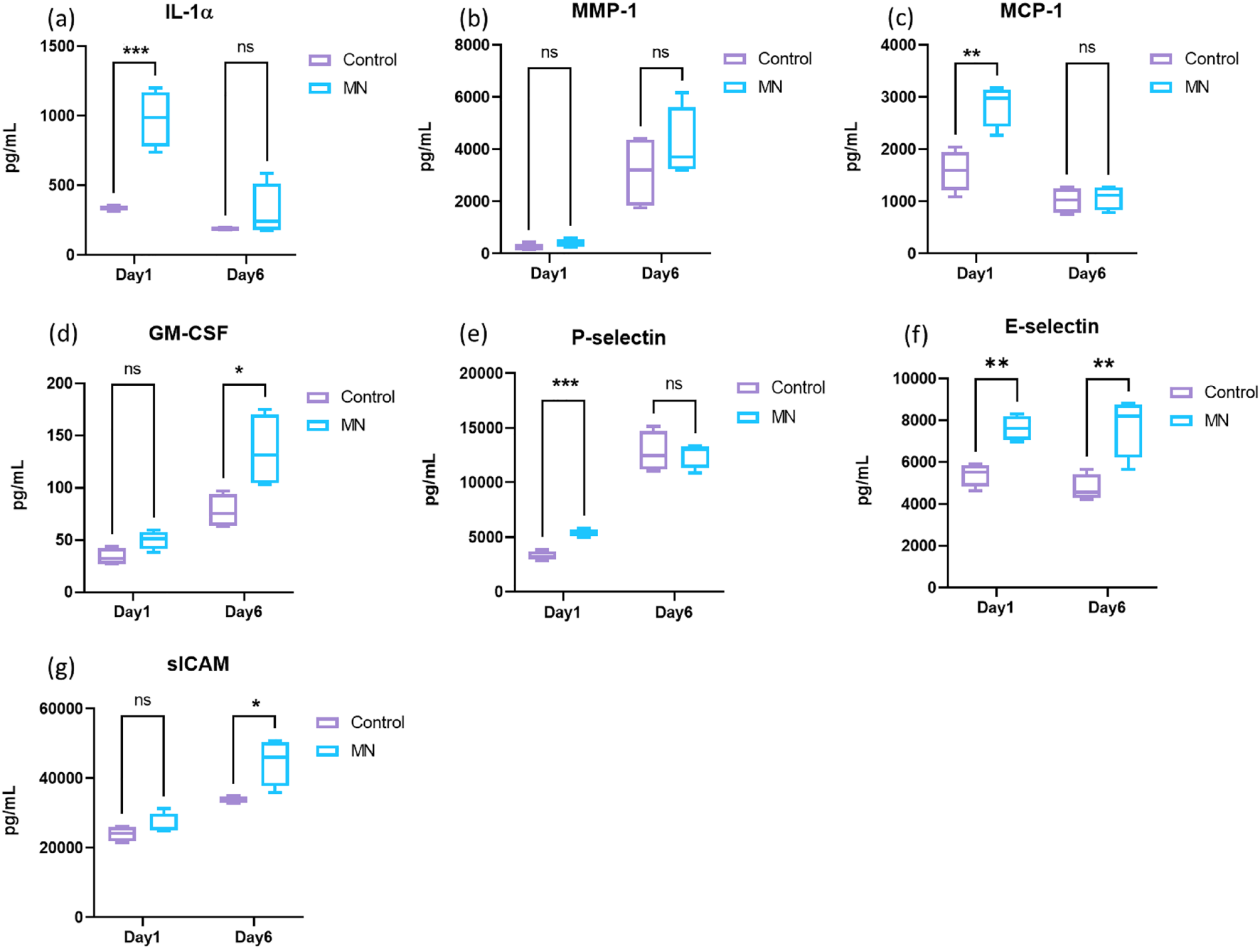
Quantification of pro-inflammatory cytokines in the ex vivo tissue. (**a**–**g**) The expression of growth factors was measured using a multiplex cytokine array. The media was collected on Day 1 and Day 6 (N = 4, 2 biological samples per donor, 2 donors). Student t-test, *P ≤ 0.05, **P ≤ 0.01, ***P ≤ 0.001, ****P ≤ 0.0001.

To demonstrate that this model carries an important component of cell–cell cross talk in early stages of wound healing, select growth factors, cytokines, and chemokines involved in the repair mechanism were analyzed by studying the tissue culture supernatant (Figs. 3, 4). Growth factor analysis reveals the microneedling procedure stimulated significant upregulation of vascular endothelial growth factor (VEGF), platelet derived growth factor (PDGF), fibroblast growth factor (FGF), and insulin (Fig. 3a–d). The activation of the above proteins typically is representative of early stages in tissue repair. Interestingly, the secretion level of transforming growth factor beta (TGF-β, Fig. 3e) was significantly higher in the microneedling treated tissues on Day 6 of culture, but not within 24 h of culturing. In addition, tumor necrosis factor (TNF-α) has been reported to be highly involved in the early process of wound healing in animal study^19^. Here, a significant upregulation in the MN treated group on Day 1 was observed, followed by decreasing to baseline level at Day 6 (Fig. 3f).

Cytokine analysis revealed a time-dependent response of release from the tissue subjected to microneedling stimuli. The expression of IL-1α, peaked 1 day after wounding while angiogenesis associated chemokines such as P-selectin, E-selectin, granulocyte–macrophage colony-stimulating factor (GM-CSF), and soluble intercellular adhesion molecule-1 (sICAM-1) continued to increase over the course of 6 days (Fig. 4). On the other hand, matrix metalloproteinase 1 (MMP-1) expression was trending higher on Day 6 compared to Day 1 (Fig. 4d) but was not found to be statistically significant in this model. In addition, the expression of IL-17A, IL-4, IL-10, IL-13 and IP-10 was below the detection limit and no significant changes were observed. Gene expression analysis revealed the MN treated group has slightly increased expression of PPARdelta (PPRD) compared to the control on Day 6 (Supplementary Fig. 2).

## Discussion

Microneedling is a popular option within the aesthetic procedure portfolio due to its minimal downtime and availability for use at both a home and professional level^6^. It is demonstrated to have a wide array of target applications, from skincare to hair-loss^20^. Clinical evaluations have shown efficacy in targeting a broad range of applications in many skin types, particularly in photoaging, acne scarring, wrinkles, dyspigmentation, and overall skin appearance^21–25^. A majority of patients who underwent microneedling treatment for acne scarring demonstrated clinical improvement by at least two grades according to the Goodman and Baron acne scar grading system^26–28^.

Ogunjimi et al. reported in a 111 subject study that the microneedling procedure creates transient pores on the surface of the skin, leading to an increase in TEWL and decrease in skin impedance with an average micropore closure time between 2 and 4 days^10^. The micropore closure time was significantly longer in the subjects with darker skin types compared to the Asian and Caucasian subject groups. Further insights into how microneedling works has been described in animal models and clinical studies^15,29–31^. A rat model has demonstrated that the inflammatory response is implicated in the healing process following microneedling through increased levels of TGF-β for up to 2 weeks post-procedure^29^. In addition, there was a measurable increase in epidermal thickening, gene expression of FGF7, Collagen I & III, as well as enhanced levels of Collagen I & III and GAGs^30^. Additional insights to the molecular mechanism of microneedling was provided by Schmitt et al. by exposing 3D reconstructed skin to microneedling stimuli^15^. This model reported an increase in the level of Keratin 13, CCL11, IGF, TIMP3 and Collagen III & VIII at 5 days post-microneedling treatment^15^. Interestingly, the gene expression of many pro-inflammatory markers such as IL-1α, 1L-1β, IL-36, IL-4 and S100 were markedly decreased in this model^15^.

A typical wound healing process of the skin proceeds via an overlapping pattern of phases including inflammation, epithelialization, formation of granulation tissue, and tissue remodeling. The crosstalk between different types of skin cells and the underlying paracrine growth factor signaling are essential to coordinate wound healing-associated processes such as proliferation and migration^32–34^. The wound healing process in ex vivo skin subjected to different physical stimuli, such as incisions, burning, laser treatment or punch biopsies, has been widely studied; these have demonstrated the ability of the tissue to re-epithelialize and modulate both growth factor and inflammatory cytokine release throughout its culture period to heal^35–38^. This ability can be attributed to the unique features of ex vivo skin. One of the most notable features is presence of all cell types, including detectable levels of immune cells up to 10 days in culture, and its ability to maintain a robust skin barrier throughout culture^39^. Additionally, the use of ex vivo skin permits for studying the effect of different donor profiles (age/sex/ethnicity) and reduces ethical constraint when testing, as compared to animal or clinical evaluations. However, the tissue is subjected to several limitations, such as accessibility, culture period time or amount of tissue received from the donor. Despite these limitations, the unique characteristics of ex vivo skin tissue, especially when compared to 2D or reconstructed human skin equivalents, enable a strong pre-clinical understanding of the wound healing process through the discovery of mechanism of action of different physical or chemical stimuli and prove to be a useful tool in dermatological research^38,40^.

The current ex vivo skin study took a comprehensive approach to understand the skin’s stimulated molecular response according to different microneedling treatment conditions. Post-microneedling, the ex vivo skin showed an immediate reduction in skin impedance and the ability to heal in all skin donors over the 6 day culture period. These effects in the microneedled tissue were appreciable despite all tissues undergoing physical manipulation via the abdominoplasty procedure, removal of the hypodermic tissue, and creation of biopsy explants, which may have induced a systemic increase in inflammatory molecules across all samples. To our knowledge, there was no difference in response based on the different skin types. This understanding reflects the common standard practice of the microneedling procedure and indicates the importance of leveraging the skin healing process post treatment. In this study, the skin showed an increase in proliferation (Ki67) and skin barrier biomarkers (TGM-1 and Filaggrin), which demonstrated that enhancing biomarkers related to barrier renewal was part of the skin’s robust response to microneedling stimuli. The ex vivo skin demonstrated a textbook like repair process, as demonstrated in the evaluation of over 20 growth factors and pro-inflammatory cytokines at 1 day and 6 days post-microneedling treatment, of a synchronized event between pro-inflammatory cytokines (immediate upregulation of TNF-α and IL-1α & β, TGF-β and MMP-1 at Day 6) with pro-angiogenic factors (enhanced level of VEGF, PDGF and FGF at both time points).

There is a great opportunity to enhance the aesthetic outcome immediately after and the ensuing days post-procedure by pairing microneedling with different actives due to improved skin penetration of cosmetic actives^41–43^. Antioxidants such as vitamin C, used in conjunction with microneedling, have been investigated for treatment of acne scars and melasma^44^. In addition, the use of topical platelet rich plasma with the microneedling procedure has shown to achieve greater efficacy compared to microneedling alone for skin rejuvenation, acne scars, and hair loss applications^44–46^. Another clinical study indicated that combining the use of microneedling treatment with 35% glycolic acid significantly improved the appearance of acne scars^47^. In general, patients are advised to use a non-allergic moisturizer and physical sunblock in order to protect the compromised skin after the procedure^6^.

Our work provided additional levels of insight to 3D reconstructed skin and rat models by demonstrating a transiently increased behavior of some of the classic early inflammatory markers. Some of the fast-responding markers peaked at day 1 and progressively decreased by 6 days following microneedling, whereas the biomarkers related to matrix remodeling increased in later time points. The molecular response observed in our model aligned more closely with the rat models published by Aust et al. and less with the 3D reconstructed skin model^15,29,30^. The difference can potentially be attributed to the difference in skin models as well as timing post microneedling treatment.

## Conclusion

In summary, the current study created a non-animal, physiologically relevant ex vivo model to further understand the mechanism of action of microneedling. This model also reflects the dynamic process of wound healing and reinforces the current clinical understanding of the microneedling procedure. We believe this model can be a significant platform for future discovery of actives that can better augment the microneedling outcome and standard of care for patients and consumers.

## Supplementary Information

Supplementary Information.

## Data Availability

The datasets used and/or analyzed during the current study are available from the corresponding author on reasonable request.
