# Supplementary material for: Utilization of ex vivo tissue model to study skin regeneration following microneedle stimuli
Source: Sci Rep. 2022 Oct 27;12:18115. doi: 10.1038/s41598-022-22481-w (PMC9613915; doi:10.1038/s41598-022-22481-w)
Supplement: Supplementary file 1 — Supplementary Information. [file 41598_2022_22481_MOESM1_ESM.docx]

**Supplementary Data**
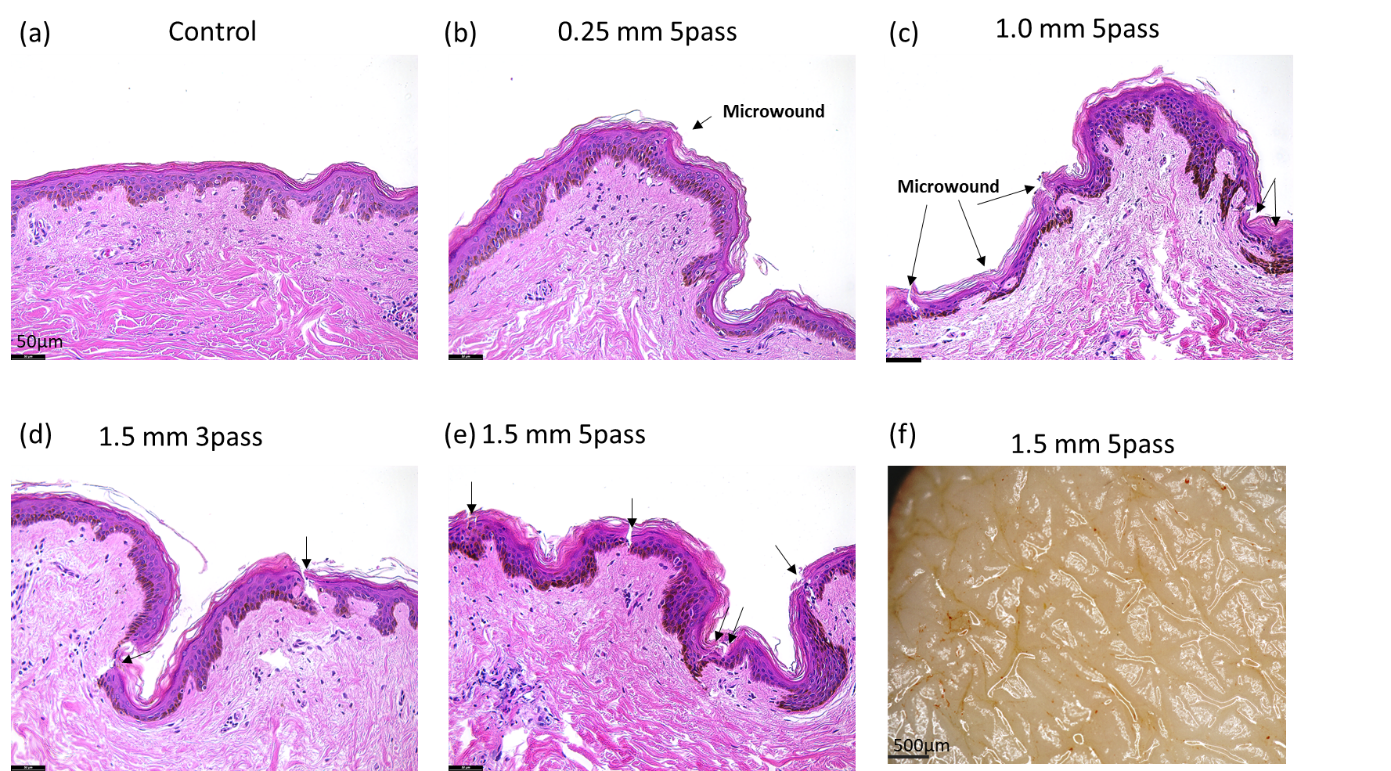


**Supplemental figure 1. Representative hematoxylin and eosin(H&E) stained sections of *ex vivo* skin tissue right after the microneedling procedure.** **(a)** H&E staining of non-treated control tissue. **(b)**H&E staining of microneeded tissue treated with 5 passes of 0.25 mm and **(c)** 1.0 mm needle lengths. **(d)** H&E staining of microneeded tissue treated with 1.5 mm needle length with 3 pass and **(e)** 5 pass. Scale bar = 50µM. **(f)** Pinpoint bleeding of tissue treated with 5 pass microneedling procedure at 1.5mm needle length. Scale bar = 500µM.


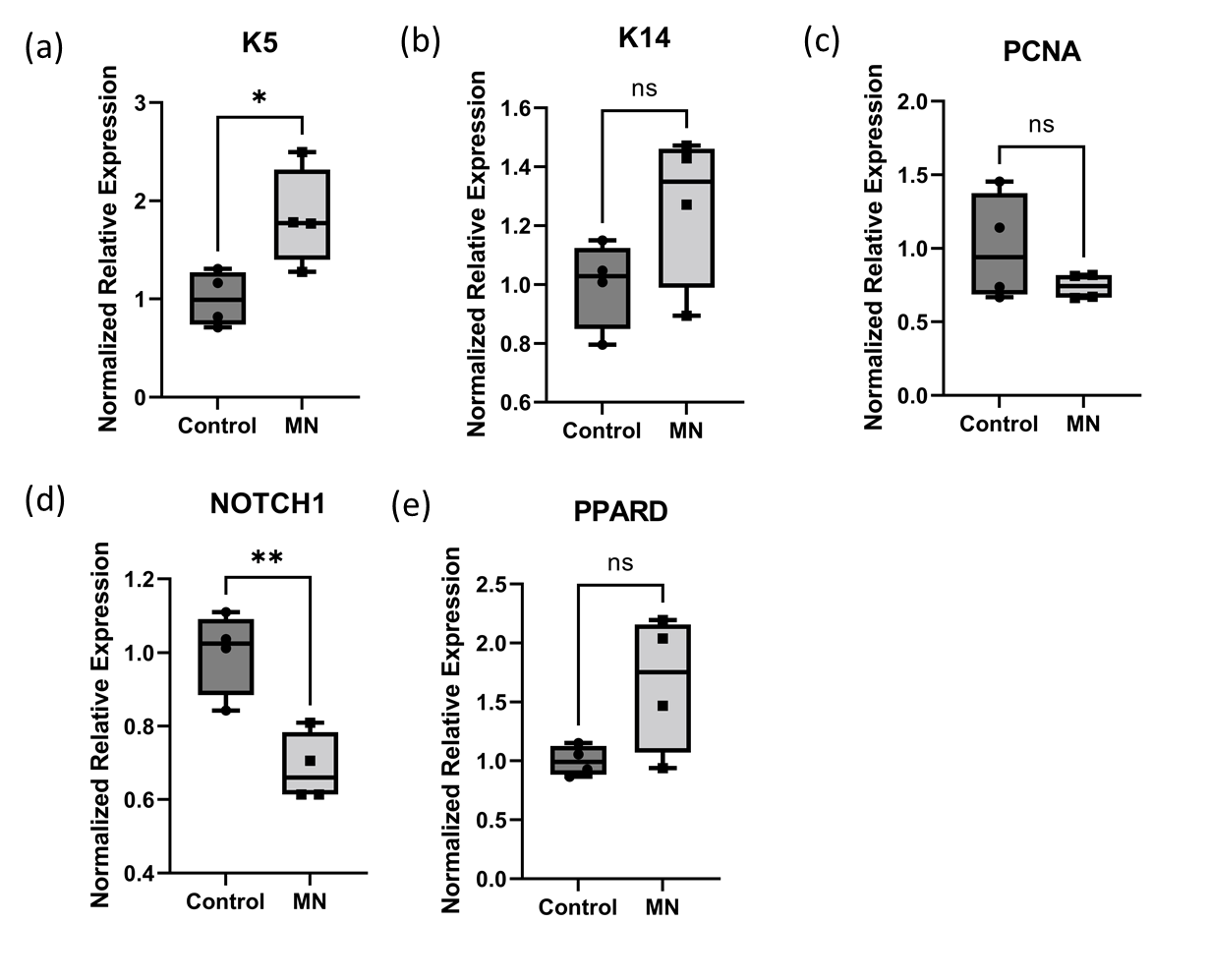


**Supplemental figure 2. Normalized relative gene expression of skin tissue.** **(a-e)** Expression of Keratin 5(K5), Keratin 14(K14), Proliferating Cell Nuclear Antigen(PCNA) , Notch homolog 1(NOTCH1) and Peroxisome proliferator-activated receptors delta(PPDARD). The data are relative to the control and to the reference gene PPIA. Data are shown as 3 individual biological samples. Student t-test, **P* ≤ 0.05, ** *P* ≤ 0.01, *** *P* ≤ 0.001, **** *P* ≤ 0.0001.
